# Effects of PPAR****γ**** Agonist Pioglitazone on Redox-Sensitive Cellular Signaling in Young Spontaneously Hypertensive Rats

**DOI:** 10.1155/2013/541871

**Published:** 2013-12-19

**Authors:** Ima Dovinová, Miroslav Barancik, Miroslava Majzunova, Stefan Zorad, Lucia Gajdosechová, Linda Gresová, Sona Cacanyiova, Frantisek Kristek, Peter Balis, Julie Y. H. Chan

**Affiliations:** ^1^Institute of Normal and Pathological Physiology, SAS, Sienkiewiczova 1, 813 71 Bratislava, Slovakia; ^2^Institute for Heart Research, SAS, Dubravska cesta 9, 840 05 Bratislava, Slovakia; ^3^Institute of Experimental Endocrinology, SAS, Vlarska 3, 833 06 Bratislava, Slovakia; ^4^Center for Translational Research in Biomedical Science, Kaohsiung Chang Gang Memorial Hospital, 123 Ta Pei Road, Kaohsiung 83301, Taiwan

## Abstract

PPAR**γ** receptor plays an important role in oxidative stress response. Its agonists can influence vascular contractility in experimental hypertension. Our study was focused on the effects of a PPAR**γ** agonist pioglitazone (PIO) on blood pressure regulation, vasoactivity of vessels, and redox-sensitive signaling at the central (brainstem, BS) and peripheral (left ventricle, LV) levels in young prehypertensive rats. 5-week-old SHR were treated either with PIO (10 mg/kg/day, 2 weeks) or with saline using gastric gavage. Administration of PIO significantly slowed down blood pressure increase and improved lipid profile and aortic relaxation after insulin stimulation. A significant increase in PPAR**γ** expression was found only in BS, not in LV. PIO treatment did not influence NOS changes, but had tissue-dependent effect on SOD regulation and increased SOD activity, observed in LV. The treatment with PIO differentially affected also the levels of other intracellular signaling components: Akt kinase increased in the the BS, while **β**-catenin level was down-regulated in the BS and up-regulated in the LV. We found that the lowering of blood pressure in young SHR can be connected with insulin sensitivity of vessels and that **β**-catenin and SOD levels are important agents mediating PIO effects in the BS and LV.

## 1. Introduction

Blood pressure is regulated by several internal systems. Power-spectral analysis identifies oscillations in heart rate and blood pressure that are modulated by inputs from the renin-angiotensin system, sympathetic and parasympathetic neurons, and locally released vasoactive factors such as nitric oxide (NO). Current findings support the view that the reactive oxygen species (ROS) and antioxidant enzymes such as superoxide dismutase (SOD) expressed in the central nervous system (CNS) play an important role in the regulation of blood pressure, and perturbations in redox homeostasis contribute to pathogenesis of hypertension [1]. Activity of ROS sources (such as NADPH oxidases), stimulated through angiotensin II system and AT1 receptors are involved in redox-sensitive intracellular signaling modulated by kinase cascades and transcriptional factors in cardiovascular system [2]. Antioxidant response and the potential sites of internal antioxidants of different SOD isoforms, could modulate blood pressure in the vasculature, the brain, and the kidney [1, 2].

The peroxisome proliferator-activated receptor *γ* (PPAR*γ*) is a nuclear receptor that takes part in the regulation of lipid metabolism and in cellular signaling. Dysregulation in the PPAR*γ* activity may underlie diseases connected with the metabolic syndrome and hypertension. As a nuclear receptor, PPAR*γ* acts together with another nuclear receptor, the retinoid X receptor, to dissociate corepressor and recruitment of coactivator protein, which, in turn, promotes transcription of the downstream target genes involved in adipocyte differentiation, glucose homeostasis, lipid trafficking, or anti-inflammatory response [3]. The activity of PPAR*γ* is influenced by a variety of extracellular ligands: glitazones (rosiglitazone, pioglitazone, and troglitazone) and intracellular ligands: prostaglandins, leukotrienes, and *α*-lipoic acid [4].

Studies of PPAR*γ* document a larger increase in blood pressure in endothelial PPAR*γ*, null mice in response to angiotensin II infusion [5]. This effect is connected with impaired vascular relaxation in response to acetylcholine combined with unaffected relaxation in response to sodium nitroprusside. These findings indicate that endothelial PPAR*γ* regulates vascular NO production and that the disruption of endothelial PPAR*γ* contributes to endothelial dysfunction *in vivo*. Oral intake of rosiglitazone (RSG) resulted in vasodepression and reduction of the augmented sympathetic vasomotor activity in the spontaneously hypertensive rat [6]. Overexpression of PPAR*γ* and amelioration of oxidative stress in the brainstem rostral ventrolateral medulla (RVLM), where sympathetic promotor neurons reside, underlies the cardiovascular protective action of RSG. A recent study showed different effects of RSG application in young versus adult SHR. While in young SHR this PPAR*γ* agonist influences PI3K/Akt/NO signaling in blood vessels through deactivation of the insulin resistance pathway, this effect was not evident in adult animals [7]. These observations open the possibility of differential effects of the PPAR*γ* agonist in cellular signaling involved in development of hypertension in SHR of different ages.

Application of the PPAR*γ* agonist pioglitazone (PIO) was found to influence the vascular contractility of blood vessels in SHR [8]. Several recent studies also demonstrated the implication of PPAR*γ* in oxidative stress responses and in the imbalances between pro-oxidant and antioxidant responses that influence apoptotic or necrotic cell death [3]. PPAR*γ* may directly modulate activation of several antioxidants involved in oxidative stress, such as the mitochondrial manganese SOD (MnSOD) [9], mitochondrial uncoupling protein (UCP2) [6], or catalase [10, 11]. This modulation was observed in several kinds of cells including cardiomyocytes [9], neuronal cells [6], adipocytes, and endothelial cells [10, 11]. The PPAR*γ* modulation also plays a role in the isoform-specific modulation of expression of NO synthases (NOSs). PPAR*γ* ligands promoted expression of endothelial NOS (eNOS) *in vivo* [5], but down-regulated the inducible NOS (iNOS) isoform [12, 13]. Moreover, the effects of PPAR*γ* ligands were found to be connected with downregulation of the inducible cyclooxygenase-2 (COX-2) [12].

In the regulation of cellular response, the modulation of these enzyme systems is associated with several signaling pathways, such as Nrf2 (antioxidant pathway), WnT/*β*-catenin (antioxidant pathway), or NF-*κ*B (prooxidant pathway) [3]. Several studies have shown the modulation of Akt kinase phosphorylation by PIO [14–17]. PIO was found to increase Akt phosphorylation (activation) [14, 15], but in some cell types also the inhibition or reduction of Akt-1 activity by PPAR*γ* agonists has been observed [16, 17]. Recently, it was reported that the protein whose function could be regulated by Akt kinase-mediated phosphorylation at Ser552 is *β*-catenin [18]. Under normal cellular conditions, *β*-catenin acting through Wnt signaling is involved in cell proliferation and differentiation, but under changed ROS conditions, its function can shift to regulate the transcription factors that support cell survival through increased stress resistance and ROS clearance [19]. This suggests that *β*-catenin can play a pivotal role in the re-programming of the transcriptional activity in response to changes in ROS [19].

Arterial blood pressure can be influenced through several main regulatory systems located in different organs and tissues: brain (mainly in brainstem), heart (ventricles), large and small vessels (such as aorta and mesentery artery), and kidney (juxtaglomerular apparatus). In several studies, the effects of PPAR gamma on blood pressure have been studied. However, these studies did not focus on redox cellular signaling in the heart and only few results were obtained in the brain [6]. Therefore, our study was focused on the investigation of PPAR*γ* agonist PIO on blood pressure regulation in young prehypertensive rats (SHR) and on redox-sensitive cellular signaling within systems of central (brainstem) and peripheral (left ventricle) regulation of blood pressure.

The concrete aims of this study included the determination of PIO effects on

1. blood pressure modulation, lipid profile, adipocytes RAS components, and vessel responses,
2. different components of redox-sensitive intracellular signaling (SOD, NOS, Akt kinase, and *β*-catenin) in the brainstem and in the left ventricle.

## 2. Materials and Methods

### 2.1. Experimental Model and Treatment Protocol

Young male spontaneously hypertensive rats (SHR; 5 weeks old, *n* = 7) were treated with PPAR*γ* agonist pioglitazone (PIO), in the dose of 10 mg/kg/day. PIO was dissolved in saline and administered orally by gavage as a suspension during 2 weeks. Animals of the control group (*n* = 7) were gavaged daily with physiological solution (saline). The body weight, daily food intake, and tap water intake were measured before, during, and after the two week treatment period. All animal experiments were performed in accordance with the rules of the State Veterinary Administration of the Slovak Republic and with the guidelines of the Animal Research and Care Committee of the Institute of Normal and Pathological Physiology of the Slovak Academy of Sciences.

### 2.2. Blood Pressure Determination

Systolic blood pressure was measured noninvasively by tail cuff plethysmography in both the control and PIO-treated groups of rats. Blood pressure measurements were performed on days 1, 5, 9, and 12 of the treatment period.

### 2.3. Collection of Samples

At the end of the experiment, the animals were sacrificed and hearts and brain were rapidly excised. Aorta was also isolated and used for further functional studies. Excised hearts were weighed, and left and right ventricles were separated. The whole heart and ventricular weights were registered. Further processing of tissue samples depended on the specific assay. For measurement of superoxide levels, tissues were collected into ice-cold Krebs buffer. For measurements of NOS and SOD activities, the tissue samples were cooled in Tris-HCl supplemented with a protease inhibitor cocktail and further processed according to individual methods. The tissue samples for molecular-biological (qPCR) and biochemical (Western blot) analyses were frozen in liquid nitrogen and stored at −80°C until further use.

Plasma samples were prepared from whole blood collected into Na-EDTA solution. After centrifugation for 5 min at 1200 ×g, the plasma was collected. Prepared plasma samples were stored at −80°C until further analysis.

### 2.4. Functional Studies *In Vitro*

Thoracic aorta was isolated, cleaned of connective tissue, and cut into rings (4 mm in length). The rings were fixed between two stainless wire triangles in an incubation organ bath containing Krebs solution (NaCl 118 mM, KCl 5 mM, NaHCO_3_ 25 mM, MgSO_4_ 1.2 mM, KH_2_PO_4_ 1.2 mM, CaCl_2_ 2.5 mM, glucose 11 mM, CaNa_2_EDTA 0.032 mM). The solution was oxygenated with pneumoxide (95% O_2_, 5% CO_2_) at 37°C. The upper wire triangles were connected to electromechanical transducers (Experimetria) and the changes in isometric tension were registered using AD converter and Dewetron software. The resting tension was adjusted to 1 g and applied to each ring.

Subsequently, preparations were equilibrated for 60 minutes and the presence of functional endothelium was assessed in all preparations. Contractile responses induced by KCl (100 mmol/L) were used to achieve receptor-independent maximal contraction. The relaxant responses were followed on rings precontracted with noradrenaline (10^−6 ^mol/L) to produce a stabile plateau of contraction. The rings were then exposed to acetylcholine (10^−5^ mol/L) or insuline (10^−6 ^mol/L) to induce a maximal response. Relaxation was expressed as a percentage of noradrenaline-induced contraction.

### 2.5. Gene Expression Determination

Total RNA from the brainstem and left ventricle tissue samples was isolated with TRIsure reagent (Bioline) according to the manufacturer's protocol. Total RNA from adipose tissue was isolated by RNeasy Universal Plus Mini Kit (Qiagen). The isolated total RNA was quantified spectrophotometrically at 260/280 nm using Nanodrop. Reverse transcription (RT) reaction was performed using TetrocDNA kit (Bioline) on Eppendorf Mastercycler.

Real-time polymerase chain reaction (qPCR) by amplification of cDNA was performed on a Biorad CFX96 Real-time system using the SensiFAST SYBR No ROX kit (Bioline). PCR reaction for each sample was carried out in duplicate for all cDNA and for the housekeeping gene of glyceraldehyde-3-phosphate dehydrogenase (GAPDH).

The primer pairs used for amplification of studied genes (PPAR*γ*, SOD1, SOD2, SOD3, NOS1, NOS3, p22phox, AT1R, UCP-1, AGT, ACE, aP2, GAPDH, and RPS29) are listed in Table 1.

### 2.6. Determination of Total Superoxide Dismutase and Nitric Oxide Synthase Activities

The total superoxide dismutase (SOD) activity was analyzed using the SOD Assay kit (Fluka) in tissue samples. The rate of WST-1 reduction with superoxide is linearly related to the xanthine oxidase activity, and is inhibited by SOD. The SOD activity was determined as an inhibition activity by measuring the color decrease of WST-1-formazan production at 450 nm (Thermo Scientific Multiscan FC).

Total activity of nitric oxide synthases (NOSs) was determined using measurement of the conversion of radioactive ^3^H-L-Arginine to ^3^H-L-Citrulline [20] and the product was detected by liquid scintillator Tri-Carb 2910 TR (Perkin Elmer).

### 2.7. Electrophoresis and Immunochemical Western Blot Analysis

Samples of protein fractions containing equivalent amounts of proteins per lane were separated by sodium dodecyl sulfate-polyacrylamide gel electrophoresis (SDS-PAGE). After electrophoretic separation, proteins were transferred to a nitrocellulose membrane. The quality of the transfer was controlled by Ponceau S staining of nitrocellulose membranes after the transfer and protein loading by using glyceraldehyde-3-phosphate dehydrogenase (GAPDH) as a housekeeper. Specific anti-SOD1, anti-SOD2, anti-Akt kinase, anti-*β*-catenin, anti-GAPDH (all from Santa Cruz Biotechnology), and anti-phospho-Akt kinase (Ser473 and Thr308) (from Cell Signaling Technology) antibodies were used for the primary immunodetection. Peroxidase-labelled anti-rabbit immunoglobulin (Cell Signaling Technology) was used as the secondary antibody. Bound antibodies were detected by the enhanced chemiluminescence (ECL) detection method.

### 2.8. Lipid Profile, Glucose and Insulin Level Determination

Plasma insulin levels were evaluated by RIA kits (Millipore) following the manufacturer's protocol. Lipid profile and blood glucose determinations were done at Synlab (Bratislava, Slovakia) using the COBAS Integra 800 multianalyzer (Roche).

### 2.9. Statistical Evaluation

The data for blood pressure measurements were evaluated using a two-way ANOVA and for *in vitro* functional studies using a one-way ANOVA with Bonferroni adjustment in post hoc tests. Data from other measurements were analyzed either by the Student's *t*-test or the Wilcoxon-Mann-Whitney *U*-test. Differences were considered significant at *P* < 0.05 in all tests.

## 3. Results

### 3.1. Blood Pressure and General Physiological Parameters

**Figure 1:**
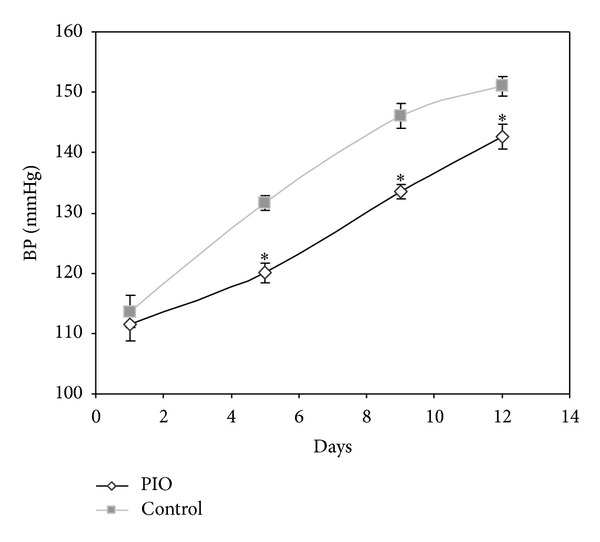
Effect of pioglitazone (PIO) administration on blood pressure of young SHRs. Blood pressure was measured using the plethysmography tail-cuff method on days 1, 5, 9, and 12 of the treatment period. The data represent mean ± SEM, *n* = 7. *P < 0.05, PIO versus control.

The blood pressure in young SHR significantly increased from 113 mmHg to 151 mmHg between the 5th and 7th week of age. Administration of the PPAR*γ* agonist PIO significantly slowed down the development of blood pressure increase in young prehypertensive animals (Figure 1).

The effects of PIO treatment on rat body weight, heart weight, the weights of the left (LV) and the right (RV) ventricles, and plasma insulin and glucose concentrations were measured and determined. The body weight and weight of the total cardiac mass were not changed significantly in PIO-treated rats. In addition, the ratios of the weights of the whole heart or ventricles versus body weight were not influenced by PIO. In young SHR treated with PIO, we observed a slightly increased weight of epididymal fat (control group: 0.31 ± 0.04 g; PIO group: 0.38 ± 0.03 g) and adiposity index (control group: 0.26 ± 0.04%; PIO group: 0.32 ± 0.02%), but these changes were not statistically significant. The treatment also did not significantly influence the plasma concentrations of insulin and glucose.

### 3.2. Vasoactivity of Vessels

In functional *in vitro* studies, we investigated the vasoactivity of aorta rings in the control and PIO-treated groups of young SHR (Table 2). Maximal relaxation of aorta was observed either after acetylcholine (Ach) or insulin (Ins) stimulation. Endothelium-dependent relaxation to Ach did not differ between groups. We observed small relaxation response to Ins in both groups; however, the maximal response to Ins was significantly higher in the PIO-treated group compared to control group (*P* < 0.01). Contractile responses induced by stimulation of adrenergic receptors (noradrenaline) as well as by depolarisation (KCl) were similar in both groups.

### 3.3. Effect of Pioglitazone Administration on Changes in Lipid Profile

**Figure 2:**
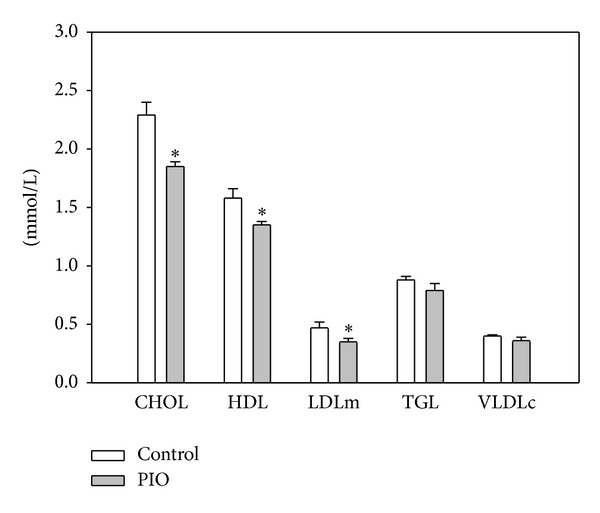
Effect of pioglitazone administration on plasma lipid profile in young SHRs. CHOL: cholesterol; HDL: high density lipoprotein; LDLm: low density lipoprotein (measured value); TGL: triglycerides; and VLDLc: very low-density lipoprotein (calculated value). Data represent mean ± SEM, *n* = 7. *P < 0.05, PIO versus control.

The observed effects of PIO on modulation of blood pressure and functional properties of vessel vasoactivity (relaxation of thoracic aorta) could reflect also changes in circulating lipids. We observed that pioglitazone treatment affected the plasma lipid profile in our young SHR. Total cholesterol concentration was significantly reduced after PIO treatment (Figure 2) and, due to the total cholesterol reduction, also HDL and LDL were significantly reduced (actually, LDL/HDL and cholesterol/HDL ratios were unchanged). Plasma triglycerides and VLDL were not influenced by the pioglitazone treatment.

### 3.4. Gene Expression in Adipose Tissues

**Figure 3:**
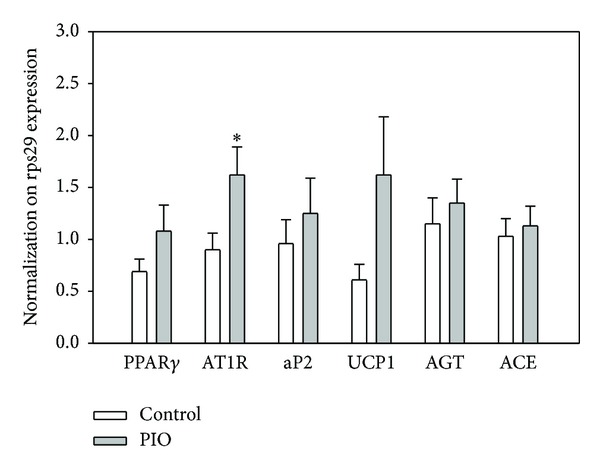
Effect of pioglitazone administration on expression of several genes in epididymal fat (adipose) tissue of young SHRs. Expression was normalized to the expression of rsp29.

mRNA expression of PPAR*γ* and AP2, markers of adipogenesis, was not changed by pioglitazone. When analyzing the mRNA of selected components of local adipose tissue RAS (AGT, ACE, and AT1 receptors), only the AT1 receptor was found significantly elevated. Gene expression of UCP1 tended to increase under pioglitazone treatment (Figure 3).

PPAR*γ*: peroxisome proliferator-activated receptor; AT1R: angiotensin-1 receptor; aP2: adipose fatty acid-binding protein; UCP1: uncoupling protein-1; AGT: angiotensinogen; ACE: angiotensin converting enzyme. Data represent mean ± SEM, *n* = 7. *P < 0.05, PIO versus control.

### 3.5. Effect of Pioglitazone on PPAR*γ* Expression in the Left Ventricle and Brainstem

**Figure 4:**
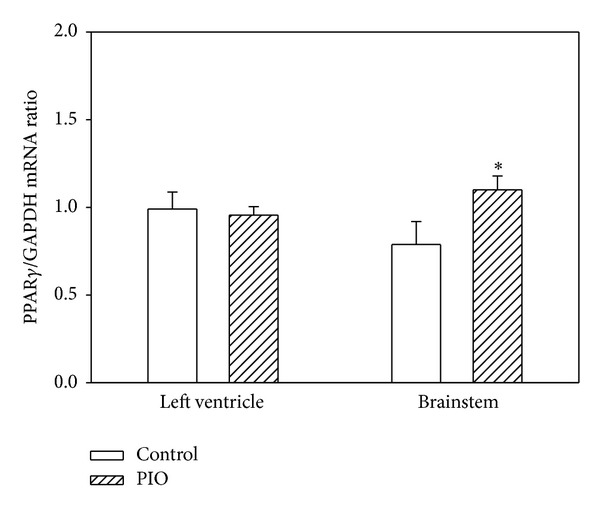
Effect of pioglitazone administration on PPAR*γ* expression in different tissues of young SHRs. PPAR*γ* expression was normalized to expression of GAPDH as a housekeeper gene. Data are shown for left ventricle and brainstem and bars represent mean ± SEM, *n* = 7. *P < 0.05, PIO versus control.

In the brainstem of the PIO-treated animals, we observed a significant increase in mRNA encoding PPAR*γ*. In the LV, we observed no change relative to control (Figure 4).

### 3.6. ROS Activation, Antioxidant Response, and SOD/NOS Balance in the Left Ventricle and Brainstem

Our study was focused also on the effects of PIO on enzymes involved in the regulation of ROS levels (p22phox subunit of NADPH oxidase system and SOD), and we observed different responses in the brainstem and the left ventricle. We found tissue-specific changes in the expression of genes involved in radical signaling, AT1R, and p22hphox. PIO treatment modulated the expression of AT1R and p22hphox only in the brainstem and induced moderate increase in their mRNA levels (by 34% for AT1R and by 20% for p22hphox).

Antioxidant response of SOD genes and SOD/NOS balance in the left ventricle and brainstem were investigated using a study of expression of individual SOD or NOS isoforms and by determination of their total activities. In the left ventricle, we observed no change in the expression of the SOD1 (Table 3) or SOD3 (not shown) isoforms. Significant changes were found only for the expression of mRNA encoding SOD2. In the brainstem, the increase of PPAR*γ* mRNA was accompanied with the upregulation of SOD2 and downregulation of SOD1 mRNA expression (Table 3). Similar to the left ventricle, the expression of SOD3 was unchanged (not shown).

**Figure 5:**
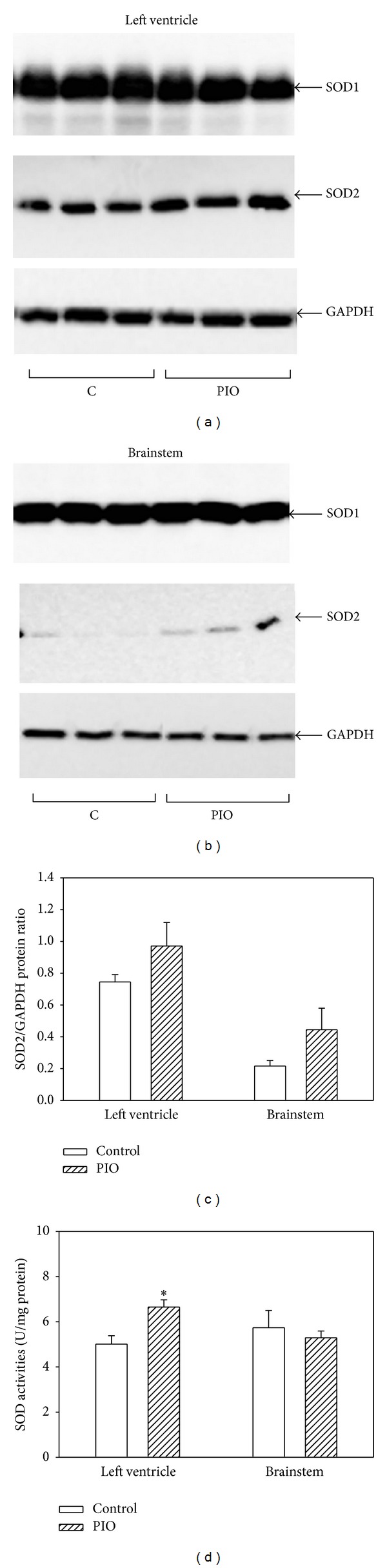
Effect of pioglitazone administration on SOD1 and SOD2 protein levels and total SOD activities in the left ventricle and brainstem of young SHRs. (a) Western blot records documenting SOD1, SOD2, and GAPDH protein levels in fraction isolated from the tissues of the left ventricle of control and PIO-treated group. (b) Western blot records documenting SOD1, SOD2, and GAPDH protein levels in brainstem. (c) Quantification of SOD2 protein levels normalized to the GAPDH protein levels. Data were obtained from Western blot records and each bar represents mean ± SEM of 7 tissue samples per group. *P < 0.05, PIO versus control. (d) The SOD activities were analyzed using the SOD Assay kit (Fluka) in tissue samples of left ventricle and brainstem. The specific activities are expressed in units per mg of proteins and are presented as mean ± SEM, *n* = 7. *P < 0.05, PIO versus control.

PIO treatment revealed different, tissue-specific effects on SOD activities. In the left ventricle, PIO induced a significant increase of total SOD activity, while no change was observed in the brainstem (Figure 5(d)). The observed increase in the total SOD activities in the left ventricle has been found to be connected with partial (nonsignificant) upregulation of SOD2 protein levels (Figures 5(a) and 5(c)). Similar changes in SOD2 protein levels were found in the brainstem (Figures 5(b) and 5(c)). Levels of the SOD1 isoform were not changed neither in LV nor in BS (Figures 5(a) and 5(b)).

**Figure 6:**
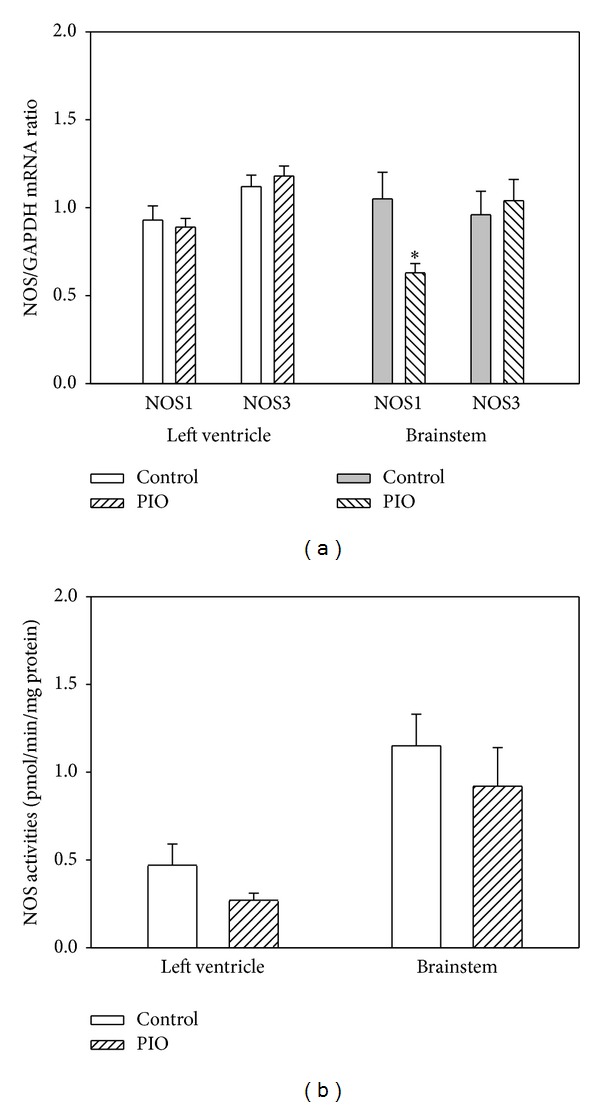
Effect of pioglitazone administration on nitric oxide synthase (NOS) expression and activities in the left ventricle and brainstem of young SHRs. (a) Expression of NOS1 and NOS3 isoforms was normalized to the expression of GAPDH. Data are shown for the left ventricle and brainstem. Bars represent mean ± SEM, *n* = 7. *P < 0.05, PIO versus control. (b) The NOS activities were determined using conversion of radioactive ^3^H-Arginine to ^3^H-Citrulline. The specific activities are expressed in pmols of citrulline produced in one min and related to 1 mg of proteins. Data are presented as mean ± SEM, *n* = 7.

For NOS isoforms, the only significant effect of PIO treatment was observed for NOS1 (nNOS), a decrease in the brainstem (Figure 6(a)). The treatment did not influence the total NOS activities in the left ventricle or in the brainstem (Figure 6(b)).

### 3.7. Effect of Pioglitazone on Regulatory Proteins Involved in Redox Signaling

**Figure 7:**
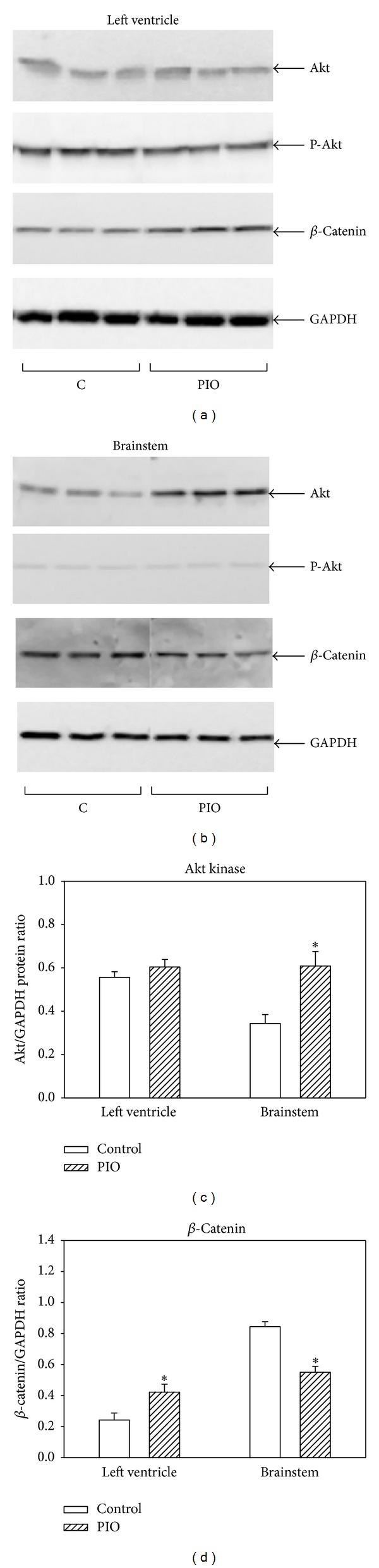
Effect of pioglitazone administration on Akt kinase and *β*-catenin in the left ventricle and brainstem of young SHRs. (a) Western blot records documenting Akt kinase, *β*-catenin and GAPDH protein levels, and levels of Akt kinase phosphorylated specifically at Ser473 (P-Akt) in protein fractions isolated from the tissues of the left ventricle. (b) Western blot records documenting Akt kinase, *β*-catenin and GAPDH protein levels, and P-Akt in brainstem. (c) Quantification of Akt kinase protein levels normalized to the GAPDH protein levels in left ventricle and brainstem. (d) Quantification of *β*-catenin protein levels normalized to the GAPDH protein levels in left ventricle and brainstem. Data were obtained from Western blot records and each bar represents mean ± SEM, of 7 tissue samples per group. *P < 0.05, PIO versus control.

The influence of PIO on Akt kinase and *β*-catenin, components of two distinct redox-sensitive signaling kinase pathways (PI3K/Akt and Wnt/*β*-catenin), was studied in tissue samples of the left ventricle and brainstem. Our data show that PIO induced increase in protein levels of Akt kinase in brainstem (Figures 7(b) and 7(c)), but not in the left ventricle (Figures 7(a) and 7(c)). Using antibodies specific for detection of Akt kinase phosphorylated specifically at Ser473 or Thr308, we looked also for changes in activation of the enzyme. However, the actions of PIO observed in the brainstem were not connected with modulation of specific Ser473 (Figure 7(b)) or Thr308 (data not shown) phosphorylation of this enzyme. We found opposite effects of PIO on *β*-catenin levels in the left ventricle and brainstem: in the left ventricle, the levels significantly increased (Figures 7(a) and 7(d)), while in the brainstem, we observed a significant decrease (Figures 7(b) and 7(d)). Protein loading was controlled by using glyceraldehyde-3-phosphate dehydrogenase (GAPDH) and we did not observe any differences.

## 4. Discussion

The development of hypertension can be induced by various stimuli and is associated with several changes in the radical and antioxidant responses as well as in cellular signaling. In the present study, we investigated the effects of PPAR*γ* agonist pioglitazone during hypertension development in young SHR. Our study focused on PIO-induced changes in the brainstem (CNS level) and the left ventricle of the heart (peripheral level) on blood pressure regulation. Moreover, the effects of PIO on changes in overall lipids and adipocytes during hypertension development in young SHR were determined.

PPAR*γ* agonists are substances used as insulin sensitizers in diabetic patients with or without hypertension [21]. Administration of PIO to young SHR in our study retarded the blood pressure development. Several studies using RSG confirmed the blood pressure lowering effect of this PPAR*γ* agonist in experimental hypertension [6, 7]. The effects of RSG observed in these studies were usually associated with changes in insulin sensitivity but without effects on glucose level. Our experimental data with PIO, another PPAR*γ* agonist, partially contradict the findings with RSG. We observed no significant change in glucose or in plasma insulin levels after PIO treatment in young rats.

In our study, SHRs were used at the age of 7 weeks, which is a period before insulin resistance is developing [22]. This was confirmed by normal glucose and insulin plasma levels in both control and PIO-treated SHR groups. In addition, plasma lipid parameters seem to be also in normal range in young SHR. However, PIO significantly reduced total plasma cholesterol by decreasing both HDL and LDL fractions. To our knowledge this is the first demonstration of PIO lipid-lowering activity in rats without dyslipidemia [23]. An explanation of the underlying mechanisms will require a study of the liver pathways of cholesterol synthesis as well as its inflammatory status in young SHR. Our *in vitro* studies of vessel vasoactivity showed improved vasodilatation response after insulin stimulation in the young SHR treated with PIO. These observations suggest increased insulin sensitivity in the aorta of treated animals and may have an impact on the delay of blood pressure increase. Similar to our data, also another PPAR gamma agonist rosiglitazone has been found to improve aortic vasodilatory response to insulin in SHR [7].

PPAR*γ* mRNA expression in LV and BS was different. While in the brainstem we obtained an increase in the gene, in the left ventricle we did not observe any changes. Therefore, we suppose that the responses in the brainstem can be directly modulated through PPAR*γ*, while in the left ventricle the regulation can be independent from PPAR*γ* and affected by pioglitazone only. Direct and indirect effects of PPAR*γ* agonists to the cardiac metabolism have been observed also on model of PPAR*γ*, knockout mice [24]. It was found that at baseline conditions, PPAR*γ* does not play a crucial role in regulating cardiac metabolism in mice. The authors suggested that it is likely due to low myocardial PPAR*γ* expression and that agonists may protect myocardium indirectly.

Some studies documented that application of RSG can activate PI3K/Akt/NOS signaling pathway in endothelium of young SHR [7]. In our study, we investigated whether PIO can affect this signaling pathway. However, in the brainstem and the left ventricle, we did not observe significant influence of PIO on Akt kinase activation. Similar to our data obtained in the left ventricle are the findings that pretreatment with PIO does not significantly increase myocardial Akt kinase phosphorylation in the rat [25]. Akt kinase expression in the brain was reported to modify food intake [26]. However, in our study, the increase in Akt kinase expression in the brainstem was not associated with significant changes in food intake and there were no differences in body weight between control and PIO-treated rats.

We also found that activities of NOS, as another component of this PI3K/Akt/NOS pathway, were not influenced by PIO. Similarly, another study showed that in an *in vivo* rat model, PIO alone did not cause significant increase in myocardial phospho-Akt or phospho-eNOS [25]. Several works investigated the association between PIO treatment and NO level. The cardioprotective effects of PIO during ischemia and reperfusion in myocardial ischemic injury were observed in rabbits and may depend on NO [27]. In patients with *diabetes mellitus*, also the effects of PIO on eNOS and iNOS were studied. In patients with insulin resistance, a significant decrease in the levels of eNOS and iNOS was observed [28]. On the other hand, these changes in NOS isoforms were not found in patients without developed insulin resistance. Our results show that NOS isoforms were not changed in the LV, while only the nNOS isoform was down-regulated in the brainstem. Moreover, PIO did not induce any change in NOS activities in young SHR. This observation may be related to the fact that no significant differences in NOS activities were observed in young hypertensive SHR and young normotensive WKY rats below 9 weeks of age. Only a tendency of a decrease in NOS activities was observed in young SHRs [29].

PIO, known as an antidiabetic agent with antioxidant and anti-inflammatory effects against oxidative stress conditions [30, 31], is also suggested to reduce, directly and/or indirectly, the overproduction of ROS. Our study was focused also on the effects of PIO on the enzymes involved in the regulation of ROS levels (p22phox subunit of NADPH oxidase system, SOD) and observed different responses in the brainstem and the left ventricle. In the brainstem, where the effects of PIO were connected to upregulation of PPAR*γ*, we found also a borderline increase in AT1 receptor, p22phox, and significant upregulation of the SOD2 isoform. The observed SOD2 upregulation is in agreement with promotor analysis showing that gene of this mitochondrial SOD isoform can be a direct target of PPAR*γ* [9]. However, total SOD activities were not influenced in the brainstem. On the other hand, there were no observable changes in AT1 receptor and p22phox in the left ventricle, and the effects of PIO were not joined directly with PPAR*γ* overexpression. In contrast to the brainstem, we observed antioxidant effects of PIO treatment in the left ventricle realized through an increase in total SOD activities. Tissue-specific differences in response to pioglitazone were observed also for *β*-catenin. Several studies have demonstrated a direct connection between PPAR*γ* signaling and *β*-catenin pathway [32–35]. Moreover, it has been shown that interaction between PPAR*γ* and *β*-catenin promotes regulation of genes that confer normal function and homeostasis to vascular cells [33]. *β*-catenin is a protein acting through Wnt signaling and under changed ROS conditions acting as a regulator of the transcription factors that support cell survival through increased stress resistance and ROS clearance [19]. The observed different effects of PIO on proteins involved in redox signaling at central and peripheral level should be clarified by further investigation.

Pioglitazone induces adipogenesis *in vitro* on 3T3L1 adipocyte culture [36]. *In vivo* RSG was shown to induce adipogenesis in subcutaneous adipose tissue [37] and this effect seems to be depot-specific [38]. In our experiment, PIO did not influence expression of PPAR*γ* and AP2, markers of adipogenesis, in epididymal adipose tissue. Thus, we conclude that stimulation of PPAR*γ* in young SHR under our experimental conditions does not lead to alternation of adipose tissue structure and mass. It has been shown that RSG decreases both angiotensinogen protein expression and angiotensin II release in isolated human subcutaneous adipocytes [39]. In addition, it was reported that PIO treatment decreases serum angiotensin II level in type 2 diabetes patients [40]. The above facts lead us to study the expression of adipose tissue RAS components under PIO treatment. Angiotensinogen and ACE mRNA in epididymal adipose tissue were not influenced by PIO, but AT1 receptor expression was significantly up-regulated. Since PIO retards the development of high blood pressure in young SHR, we hypothesize that the reduced serum angiotensin II might be involved in the mechanisms of this blood-reducing effect. Despite the absence of data on serum angiotensin II, we speculate that the upregulation of AT1 receptor expression is a physiological response to decreased serum angiotensin II.

## 5. Conclusion

Our results show that the treatment of young SHRs with PIO retards high blood pressure development, and this effect is associated with an improvement of lipid profile (CHOL, LDL) and vessel vasoactivity, without changes in glucose and insulin plasma levels, and with some changes in adipocyte RAS components. The treatment also differentially affects the redox-sensitive intracellular signaling (SOD, NOS, Akt kinase, and *β*-catenin) in the brainstem and in the heart. Lowering of blood pressure in young SHR can be directly affected by vessel vasorelaxation stimulated by insulin, and our data suggest that *β*-catenin and antioxidant SOD response, but not NOS, can be important agents of PIO effects in the brainstem and the left ventricle of young prehypertensive rats.

## Figures and Tables

**Table 1:** Primer pairs used for amplification of selected genes.

Gene	Forward (sense) primer	Reverse (antisense) primer	Amplicon size (bp)	Temp (°C)
SOD1	CAC TCT AAG AAA CAT GGC G	CTG AGA GTG AGA TCA CAC G	124	54
SOD2	TTC AGC CTG CAC TGA AG	GTC ACG CTT GAT AGC CTC	122	54
SOD3	CTT GAC CTG GTT GAG AAG ATA G	GAT CTG TGG CTG ATC GG	154	58
NOS3	CCC ACA GTC TGG TTG CT	TCA CCG TGC CCA TGA GT	124	57
NOS1	CGC TAC GCG GGC TAC AAG CA	GCA CGT CGA AGC GGC CTC TT	118	60
GAPDH	TGG AGG TGC TGG AAG AGT T	TCA CGC CAC AGC TTT CCA	103	57
p22phox	CAG GCA TAT ACC CGC TAC CT	TCT GTC ACC CTG TGC TTG AC	120	60
ACE	ATG GTA CAG AAG GGC TGG AA	TTG TAG AAG TCC CAC GCA GA	170	60
AGT	CAT GAG TTC TGG GTG GAC AA	AAG TTG TTC TGG GCG TCA CT	95	60
AT1R	TCT CAG CAT CGA TCG CTA CCT	AGG CGA GAC TTC ATT GGG TC	51	60
aP2 (FABP4)	AGC GTA GAA GGG GAC TTG GT	ATG GTG GTC GAC TTT CCA TC	185	60
PPAR*γ*	AGG ATT CAT GAC CAG GGA GTT	AGC AAA CTC AAA CTT AGC CTC CAT	79	60
RPS29	GCT GAA CAT GTG CCG ACA GT	GGT CGC TTA GTC CAA CTT AAT GAA G	74	60
UCP-1	GCC TCT ACG ATA CGG TCC	TGC ATT CTG ACC TTC ACC AC	145	60

**Table 2:** Vasoactive responses influenced by PIO treatment on relaxation and contraction of thoracic aorta.

	*n *	Control group	*n *	PIO-treated group
Ach (10^−5^ mol/L)	10	76.89 ± 6.85%	14	75.67 ± 5.8%
Ins (10^−6^ mol/L)	8	6.81 ± 2.43%	11	20.29 ± 2.48%**
NA (10^−6^ mol/L)	8	0.18 ± 0.04 g	15	0.24 ± 0.03 g
KCl (100 mmol/L)	11	0.98 ± 0.05 g	8	0.96 ± 0.03 g

Ach: acetylcholine; Ins: insulin; NA: noradrenaline; KCl: potassium chloride, **P < 0.01 PIO versus control group.

**Table 3:** mRNA expression of SOD1 and SOD2 isoforms after PIO treatment.

mRNA	Left ventricle		Brainstem	
	Control	PIO	Control	PIO
SOD1/GAPDH	1.46 ± 0.115	1.30 ± 0.087	1.39 ± 0.247	0.75 ± 0.099*
SOD2/GAPDH	1.20 ± 0.107	0.85 ± 0.075*	0.97 ± 0.067	1.24 ± 0.078*

Data represent mean ± SEM, *P < 0.05, PIO versus control.
